# Animal Anatomy Teaching: New Concepts, Innovations and Applications

**DOI:** 10.3390/vetsci13030221

**Published:** 2026-02-26

**Authors:** Om Prakash Choudhary

**Affiliations:** Department of Veterinary Anatomy, College of Veterinary Science, Guru Angad Dev Veterinary and Animal Sciences University, Rampura Phul 151103, Punjab, India; dr.om.choudhary@gmail.com or om.choudhary@gadvasu.in

Traditional methods in animal anatomy teaching, such as theoretical lectures, cadaver dissections, and prosected specimens, have long dominated the veterinary curricula. However, they are increasingly challenged by ethical issues related to animal procurement, health risks from formalin exposure, specimen scarcity, and difficulties in visualizing the complex three-dimensional structures vital for clinical proficiency across the species such as bovines, equines, canines, and avians [1,2]. New pedagogical concepts emphasize active, student-centered learning to surmount these barriers, including peer-led gamification initiatives such as the Vet Academic Challenge, in which students craft and debate exam-oriented questions on topics such as ossification in the porcine scapula or the ligamentous anatomy of the ovine stifle joint. This initiative achieved 89% engagement, heightened motivation, enhanced teamwork, and a correlation coefficient of r = 0.78 between participation and willingness to repeat, as evidenced by Likert-scale surveys and ANOVA. Flipped classroom models and problem-based learning (PBL) integrate pre-class multimedia, such as annotated dissection videos, allowing in-class time to be focused on synthesis, better supporting critical thinking and retention compared to passive lectures in gross anatomy courses. Multimodal, student-centered approaches further blend these with collaborative quizzes, encouraging holistic comprehension in accordance with veterinary competencies for diagnostics, surgery, and interdisciplinary one-health applications [3].

Technological innovations are revolutionizing anatomical instruction with precision, interactivity, and scalability. Three-dimensional (3D) printing transforms CT and MRI DICOM data into STL files for fabricating durable, odorless replicas of structures such as the canine humerus, feline pelvis, or avian wing myology using fused deposition modeling (FDM) or stereolithography (SLA), enabling tactile, repeatable exploration free from biohazards that is ideal for modular arthrology teaching. Virtual reality (VR) platforms, exemplified by Metaverse911, offer immersive haptic simulations of physiological processes—from ruminant cardiac cycles to equine respiratory mechanics—facilitating 360-degree virtual layer dissections. Augmented reality (AR) superimposes volumetric holograms on physical models to clarify neurovascular and myotendinous relationships. Artificial intelligence (AI) further advances these developments through generative tools like Nano Banana, which create breed-specific parametric diagrams (e.g., brachycephalic airway anomalies in Bulldogs), and deep convolutional neural networks that enable automated 3D reconstructions from imaging data, standardizing anatomical variations for remote asynchronous learning. Advanced techniques include Biodur E20 epoxy corrosion casts, which illuminate microvascular architectures in porcine renal or pulmonary systems and can be adapted for veterinary splanchnology, and 4D bioprinting with stimuli-responsive hydrogels that dynamically contract like myocardium or exhibit peristalsis like intestines, simulating pathophysiology for advanced training [3,4,5].

In veterinary education, these innovations converge into multimodal hybrids—integrating Anatomage Table Vet for precise Triadan dental charting in endodontics, AI-adaptive quizzes mirroring ICAR NET/JRF osteology syllabi, and haptic 3D-printed phantoms for laparoscopic simulations—yielding 25–30% improvements in visuospatial skills, procedural accuracy, empathy, and stress reduction, as shown in randomized trials among first-year Doctor of Veterinary Medicine (DVM) students. Post-COVID-19 hybrid paradigms use VR for foundational osteology priming before wet-lab validation, with longitudinal Brazilian studies indicating 61% learner preference for digital scaffolds, reduced absenteeism, and improved necropsy proficiency, proving invaluable in resource-limited contexts such as the veterinary colleges situated in rural areas. Clinical translations include VR presurgical rehearsals to curb orthopedic mishaps, 4D models forecasting fracture-implant biomechanics, generative AI for telemedicine tomographic overlays in livestock and exotics, and open-source STL repositories democratizing access to caprine orthopedics or avian coelomic layouts. These tools enhance the equity for diverse learners, from urban exam preparers to field practitioners, while AI analytics identify the curricular gaps for real-time refinement [4].

Prospective developments signal a 2027 paradigm in which metaverse consortia facilitate global virtual dissections, blockchain-secured NFTs archive rare pathologies, and quantum-boosted AI models reveal evolutionary morphometrics, all governed by ethical frameworks to mitigate dataset biases. Persistent hurdles—high initial costs, infrastructural deficits, and digital literacy of faculty—demand subsidized printer networks, targeted workshops, and decade-long cohort studies tracking board certification rates, malpractice declines, and zoonotic response efficacy. By transcending cadaver silos toward adaptive, immersive ecosystems, these advancements provide veterinarians with unparalleled anatomical mastery, welfare consciousness, and innovative acumen for precision medicine and addressing global challenges [6,7].

The five research articles published in this Special Issue collectively redefine the frontiers of veterinary education and therapeutic innovation in the era of technological advancement. These works, spanning the applications of artificial intelligence in osteology, digital anatomy visualization, active learning pedagogies, and natural compound therapeutics for pulmonary pathology, address pressing challenges in veterinary training and clinical practice. Published amid the rising enrolments and ethical sourcing pressures, these works democratize complex anatomy for global classrooms, fieldwork, forensics, and clinics, aligning with the One Health imperatives.

In the first research article, titled “Smart Osteology: An AI-Powered Two-Stage System for Multi-Species Long Bone Detection and Classification,” the author focused on the development and validation of Smart Osteology, marking a transformative milestone in veterinary anatomy education and forensic science, addressing critical barriers such as cadaver scarcity, species-specific osteological expertise deficits, and the need for accessible fieldwork tools. This pioneering two-stage AI system—combining YOLOv5 object detection (achieving 96.6% mAP@0.5) with CNN classification via ResNet34 (97.6% top-1 accuracy)—demonstrates unprecedented precision across 26,148 images of scapula, humerus, and femur from cattle, horses, and dogs. Trained on diverse, real-world datasets, the offline mobile application ensures data sovereignty for forensic applications while delivering voice-activated, PDF-exportable virtual assistance, making complex multi-species identification instantaneous and democratized.

Student validation across 150 veterinary learners yielded compelling results: 98% rated the interface user-friendly, with over 90% endorsing anatomical accuracy and overall performance. This exceptional reception underscores AI’s capacity to provide real-time feedback, known to enhance knowledge retention by up to 40%, particularly in resource-constrained global settings. By overcoming traditional limitations, such as inconsistent specimen availability and formalin exposure risks, Smart Osteology emerges as an ethical, scalable supplement to dissection, fostering visuospatial mastery essential for surgical proficiency and clinical diagnostics [8].

Beyond education, the system’s forensic utility shines in archeology and crime scene analysis, where rapid, non-destructive species identification from fragmented remains proves invaluable. Its closed-loop architecture maintains evidentiary integrity, positioning it as a gold standard for legal veterinary contexts. As the first published study in this domain (*Vet. Sci.* 2025, 12, 765), Orhan’s work from Erciyes University sets a benchmark, inviting global adoption and extension to additional bones, species (e.g., wildlife), and integration with AR/VR platforms [8].

Looking forward, Smart Osteology heralds an AI-driven era in veterinary sciences, where precision osteology becomes ubiquitous. Future enhancements involving multilingual support, expanded osteological datasets via federated learning, and hybrid models fusing detection with 3D reconstruction promise even greater utility. This innovation not only equips future veterinarians with cutting-edge tools but also accelerates One Health applications, from wildlife forensics to pandemic preparedness, ensuring anatomical expertise that transcends geographical and economic barriers [8].

The second research article is entitled “Anatomage Table Vet for Teaching the Triadan Dental Classification System: A Brief Trial and Feedback from the Students”. Complementing AI’s automation, digital dissection tools like the Anatomage Table Vet (ATV) offer immersive 3D alternatives to traditional methods. In this research article, the authors conducted a controlled trial with 89 novice students learning the Triadan Dental Classification System (TDCS) and compared the use of the ATV to traditional textbook study. Both groups showed significant post-test improvements (*p* < 0.01), with no performance differences; however, feedback revealed ATV’s motivational edge: 85% found it stimulating, 83% planned to use it independently, and 95% deemed it useful for anatomical review. Despite the ease-of-use concerns (only 55% agreement), the tool’s annotation system, which displays the labels on interaction, facilitated the spatial understanding of canine dentition, addressing cadaver shortages amid the rising student numbers. This aligns with the broader trends where 3D models strengthen visuospatial skills essential for surgery and diagnostics, positioning ATV as a supplementary asset rather than a replacement for dissection [9,10,11].

The third research article, entitled “Active Learning in Veterinary Anatomy Education: Investigating the Impact of Peer-Led Q&A Games and Multimedia on Student Perceptions,” highlighted that pedagogical evolution proceeds with active, student-centered strategies that amplify the technology’s impact. The authors from Universidad Cardenal Herrera investigated the CEU implemented peer-led Q&A games in veterinary anatomy, where students crafted exam-style questions during monitored tournaments, with sessions recorded as multimedia resources via QR codes. Achieving 89.33% participation, the approach garnered high satisfaction (Cronbach’s α = 0.920), with over 60% of respondents noting enhanced collaboration, motivation, and exam preparation [6]. By shifting from passive lectures to gamified interaction, this method fosters critical thinking, teamwork, and retention, the keys to stress-prone veterinary curricula, while blended resources extend learning beyond the classroom. These peer-driven models support the evidence that active engagement outperforms traditional instruction, preparing graduates for clinical integration [6,12].

The fourth research article of the Special Issue is entitled “Protective Effect of Tyrosol on BALF Cytology and Biochemistry in Rats Administered Intratracheal Bleomycin,” published by Ekinci et al. [13]. Transitioning to therapeutics, natural compounds show promise against oxidative lung pathologies prevalent in veterinary practice. In this research article, the authors explored tyrosol, a phenolic constituent from olive oil, in a bleomycin (BLM)-induced rat lung injury model, focusing on bronchoalveolar lavage fluid (BALF) cytology and biochemistry. Intratracheal BLM (4 mg/kg) elevated lymphocytes/neutrophils (up to 37%/19%), reduced macrophages (38%), and increased foamy macrophages, MDA, and IL-6, while depleting SOD/GPx/CAT. Tyrosol (20–80 mg/kg) reversed these in a dose-dependent manner: macrophage ratios rose to 81%, foamy cells declined, MDA/IL-6 dropped significantly (*p* < 0.05 at 80 mg/kg), and antioxidants recovered, confirming the anti-inflammatory/antioxidant efficacy without toxicity. This first BALF-specific study positions tyrosol as a fibrosis mitigator, relevant to BLM-like toxicities in oncology-treated animals or environmental exposures [13].

The fifth research article in the Special Issue, entitled “Interactive Mixed Reality Simulation Enhances Student Knowledge and Ultrasound Interpretation in Sheep Pregnancy Diagnosis,” reinforced translational pathology themes by extending molecular insights into veterinary diagnostics and interventions, though its specifics amplify the issue’s focus on innovative solutions. This study employed Ewe Scan, an innovative mixed reality (MR) training tool using Apple Vision Pro, to teach ultrasound-based pregnancy diagnosis in sheep to forty-two first-year veterinary students, randomized to MR or traditional lecture groups, with assessments conducted immediately post-training and after six weeks. The three-stage app delivered an introductory video, interactive 3D sheep anatomy models (empty/single/twin pregnancies) highlighting structures like placentomes and amniotic sacs, and a realistic scanning simulator where virtual probe positioning dynamically generated the ultrasound images in a farm environment. MR-trained students significantly outperformed lecture peers in comprehension and retention: immediate overall scores reached 90% (MR) vs. 66% (lecture), dropping to 83% vs. 52% at follow-up (*p* < 0.015), with ultrasound interpretation showing the largest gains (93% vs. 55% immediate; 80% vs. 39% follow-up). MR enhanced spatial reasoning for translating 2D images to 3D anatomy, boosting engagement (*p* < 0.001), confidence (*p* < 0.001), and satisfaction, with 80.5% preferring MR despite minor technical issues, whereas the lecture students cited pacing and comprehension problems. No motion sickness occurred, underscoring Ewe Scan’s ethical, scalable potential to reduce live-animal use while preparing students for clinical practice through immersive, active learning [14,15].

Collectively, these papers weave a narrative of constructive collaboration: AI and 3D tools like Smart Osteology and ATV overcome educational silos, active gamification sustains engagement, and tyrosol exemplifies bench-to-bedside translation.

This Special Issue encourages the veterinary community to embrace hybrid paradigms: AI democratizes osteology, 3D and gamified anatomy builds competence, and phenolics combat pathology. Future directions include AR-AI fusions for multi-species training, scaled Q&A platforms, and tyrosol trials in equine fibrosis. By blending tradition with innovation, we advance the One Health, cultivating veterinarians who are technically proficient, collaboratively adept, and therapeutically astute. I extend my gratitude to the authors, reviewers, and editors of the journal *Veterinary Sciences* for amplifying these voices and advancing our field.
